# Pan-Immune-Inflammation Value Predicts Disease Activity and Ocular Involvement in Behçet’s Disease: A Case-Control Study

**DOI:** 10.31138/mjr.200625.era

**Published:** 2026-03-01

**Authors:** Eman M. Shawky, Maha S. I. Abdelrahman, Sara Farrag

**Affiliations:** Department of Rheumatology, Rehabilitation and Physical Medicine, Faculty of Medicine, Assiut University, Assiut, Egypt

**Keywords:** Behçet syndrome, inflammation, blood cell count, lymphocytes, neutrophils, monocytes

## Abstract

**Objective/Aim:**

Behçet’s disease (BD) lacks distinctive laboratory findings, so this study aimed at evaluating the pan-immune-inflammation value (PIV), neutrophil to lymphocyte ratio (NLR), platelets to lymphocytes ratio (PLR), and monocytes to lymphocytes ratio (MLR) in BD in addition to investigating their relationship with disease activity.

**Methods:**

This case control study included 40 BD patients and 40 age and sex matched healthy volunteers. Complete clinical evaluation and disease activity scoring were conducted. Complete blood count was done for all participants with calculation of PIV, NLR, PLR and MLR.

**Results:**

BD patients showed significantly higher PIV, NLR, PLR and MLR level than healthy participants (p=0.04, 0.04, < 0.001 and 0.007, respectively). PIV showed significant positive correlation with BDCAF and erythrocyte sedimentation rate (p= 0.025 and 0.039, respectively). We detected a significantly greater PIV in patients with active ocular and musculoskeletal disease (p=0.001 and 0.037 respectively). At a cutoff of 238.26, PIV yielded an AUC of 0.81 (95% CI, p=0.001), with 83% sensitivity and 82% specificity for detecting active ocular involvement.

**Conclusion:**

PIV showed positive correlation with disease activity. PIV is superior to other studied blood cells derived ratios in detecting active ocular involvement in BD patients.

## INTRODUCTION

Behçet’s disease (BD) is a multiorgan vasculitis that affects both venous and arterial systems impacting blood vessels of all sizes. Mucocutaneous, articular, ophthalmic, gastrointestinal, vascular, and neurological involvements are present in this disease.^1, 2^ The main mechanism causing thrombosis in BD is the inflammation of the vascular wall. Systemic inflammation and an abnormal immune reaction change haematological and biochemical parameters.^3^

Platelets and leukocytes are important players in thrombosis and systemic inflammation. ^4,5^ Changes in blood cells derived ratios and indices have become known as significant predictors of diagnosis and disease activity and severity in rheumatologic disorders.^6,7^ Pan-immune-inflammation value (PIV), which is based on neutrophils, platelets, monocytes, and lymphocytes, was initially developed by Fuca et al., for the assessment of the inflammatory status. Fucà et al. demonstrated that PIV performed better than earlier immune-inflammatory indicators, such neutrophil to lymphocyte (NLR), in patients with colorectal malignancy.^8^ PIV has been investigated in several rheumatological conditions such as rheumatoid arthritis (RA) and vasculitis.^9,10^

Haematological laboratory tests are simple and inexpensive to perform in clinical settings. It is critical to discover which blood parameters are most efficient in predicting disease activity and tracking treatment response in BD. Despite the known utility of NLR, monocyte to lymphocyte (MLR) and platelet to lymphocyte ratio (PLR) in BD,^11^ the role of PIV as a more comprehensive inflammatory marker remains underexplored particularly in ocular involvement. Our study aimed at evaluating PIV and blood cells derived inflammatory ratios in BD as well as relating them to disease manifestations, and mainly ocular activity.

## PATIENTS AND METHODS

### Study Design and Participants

This observational case-control study recruited 80 participants with a 1:1 case control ratio. The BD group included 40 patients fulfilling the revised International Criteria for Behçet’s Disease (ICBD).^12^ The control group included 40 healthy volunteers. Participants were enrolled from the Rheumatology Outpatient Clinic, Assiut University Hospitals (January 2024–December 2024). The Sample size was estimated via Open EPI, version 3 software, on the following assumption: According to the literature, there is a large sized difference in the studied parameters levels between BD patients and controls.^13^

Eligibility criteria: Patients who fulfilled the revised ICBD.^12^

Exclusion criteria: The presence of the following conditions: smoking, alcohol use, the use of oral contraceptives, diabetes mellitus, endocrine disorders “thyroid dysfunction, adrenal insufficiency, or diabetes mellitus”, cancers, chronic hematologic illnesses, active infectious diseases, peripheral or coronary artery disease, hypertension, and other autoimmune or autoinflammatory conditions.

### Ethical Considerations

Ethical approval was obtained from the Ethical Committee and Institutional Review Board of the Faculty of Medicine, Assiut University, Assiut, Egypt (IRB No.: 042024300502), before study initiation. The study protocol adheres to the tents of Helsinki Declaration. All participants signed a written informed consent before inclusion in the study and after explaining the study’s purpose and procedures

### Study Outcomes and Potential Confounders

The study outcomes included an evaluation of PIV, NLR, MLR and PLR in BD patients in comparison to healthy participants and relating these markers to disease activity.

To minimise potential confounding and bias, controls were frequency matched with cases in terms of age and gender, all laboratory tests were performed with the same equipment, and the previously mentioned exclusion criteria were set to avoid confounding from comorbidities.

### Sociodemographic and Clinical Assessment

Every participant underwent a thorough history recording that included demographic data and comorbidities. Disease onset, disease manifestations as well as medications were recorded for all patients. Comprehensive general, rheumatological, neurological, and ophthalmic examinations were performed for BD cases.

- The Behçet’s Disease Current Activity Form (BDCAF) was applied for disease activity scores recording in the preceding four weeks. The final score ranges from 0 to 12 with higher score indicating higher disease activity.^14^
- Behçet’s Syndrome Overall Damage Index (BODI)^15^ was assessed for every patient.

### Ocular Involvement Assessment

Ocular evaluation was performed by a senior ophthalmologist as a part of standard clinical care:

1. Standardised Definitions:
  - Active Ocular Involvement: Presence of ≥1 of:
    Anterior uveitis (cell/flare in anterior chamber)
    Posterior uveitis (vitritis, retinal vasculitis on fundoscopy)
    Macular oedema (optical coherence tomography-confirmed) ^16^
  - Inactive Disease: Absence of inflammation in all ocular compartments for at least 3 months and grade 0 cells in anterior uveitis.^17^
2. Objective Metrics:
  - Visual acuity (Snellen chart)
  - Anterior chamber cell grading (SUN criteria) ^17^
  - Vitreous haze score (Nussenblatt scale) ^18^
  - Fluorescein angiography for retinal vasculitis.

### Laboratory Assessment

Laboratory assessment, conducted for cases and controls, encompassed the blood haemoglobin, red blood cell count, differential white blood cells (WBCs) count and platelets. To calculate PIV, the neutrophil count (10^3^/mL) was multiplied by the platelet count (10^3^/mL) and monocyte count (10^3^/mL), then divided by the lymphocyte count (10^3^/mL). NLR, MLR, and PLR were computed by dividing the neutrophil, monocyte, and platelet counts, respectively by the lymphocyte count. Erythrocyte sedimentation rate (ESR), measured via the Westergren method, was performed for cases only.

Patients were categorised into two groups, based on distinct clinical features, according to each of the following: presence or absence of active ocular involvement, presence or absence of active musculoskeletal involvement, presence or absence of active neurological involvement and receiving or not receiving anti-tumour necrosis factor (TNF) therapy.

### Statistical Analysis

All variables were assessed at baseline. Complete data was available for all participants across all studied variables. Data was analysed via SPSS version 26. Normally distributed data was presented as mean ± standard deviation (SD) while non normally distributed data was expressed as median and range. Categorical variables were presented as frequencies and percentages. For comparing quantitative variables, the Student T test was applied for normally distributed data. The Mann-Whitney U test was applied for non-normally distributed data including comparison between subgroups. Chi-square (χ^2^) and Exact tests were utilised to compare categorical variables. Spearman correlation was applied to assess correlations. Receiver operating characteristics (ROC) analysis was done to test the capability of PIV to distinguish patients with active ocular involvement. The P-value is considered significant at less than 0.05. All tests were 2 tailed.

## RESULTS

Initial assessment included 53 BD patients and 50 healthy subjects. Eleven patients were excluded after application of the previously mentioned exclusion criteria and two patients declined participation while, 10 healthy individuals were excluded as they met one or more of the exclusion criteria. Finally, our study included 40 BD patients and 40 healthy volunteers. No statistically significant difference was found between the two groups regarding age and sex distribution, as presented in **Table 1**. **Table 2** illustrates the clinical and laboratory features of the BD cases at the time of the study. Oral ulceration was the most common presenting feature of BD cases (65%) followed by inflammation of anterior and posterior segments of the eye (45%). As shown in **Table 3**, the most frequently administered drugs by study cases were azathioprine and corticosteroids.

**Table 1.** Sociodemographic data of the study subjects.

	**Cases**	**Controls**	**P value**
Age	34.75 ±6.8	36.2±7.56	0.37
Sex			
Males	29 (72.5%)	35 (87.5%)	
Females	11 (27.5%)	5 (12.5%)	0.094

Data expressed as mean± SD or frequency (%).

The P-value was significant if < 0.05.

**Table 2.** Clinical and laboratory evaluation of BD patients (n=40).

**Variable**	**Mean ±SD/Median (range)/ N (%)**
**Disease duration (years)**	5 (1–20)
**Oral ulcers**	26 (65%)
**Ocular manifestations**	18 (45%)
**Genital ulcers**	13 (32.5%)
**Musculoskeletal manifestations**	7 (17.5%)
**Neurological manifestations**	8 (20%)
**Vascular manifestations**	4 (10%)
**Gastrointestinal manifestations**	2 (5%)
**BODI**	6.63±2.26
**BDCAF**	3 (0–7)
**ESR mm/hr**	36.55± 19.29
**White blood cell count x 10^3^/ul**	6.38±1.85
**• Neutrophils x 10^3^/ul** **• Lymphocytes x 10^3^/ul** **• Monocytes x 10^3^/ul**	3.11 (1.34–8.08) 1.9 (0.85–4.49) 0.46±0.2
**Red blood cell count x 10^6^/ul**	4.92±0.71
**Haemoglobin (g/dl)**	13.6±1.71
**Platelets x 10^3^/ul**	284.5 (150–626)

Data expressed as mean± SD/ median (range) or frequency (%)., ESR: erythrocyte sedimentation rate, BODI: Bechet’s syndrome overall damage index, BDCAF Behcet’s Disease Current Activity Form.

**Table 3.** Current treatment of BD patients.

**Drug**	N=40
**Azathioprine**	32 (80%)
**Corticosteroids**	32 (80%)
**Colchicine**	30 (75%)
**Methotrexate**	29 (72.5%)
**Cyclophosphamide**	16 (40%)
**Adalimumab**	11 (27.5%)
**Anticoagulant therapy**	6 (15%)
**Cyclosporine**	1 (2.5%)

Data expressed as N (%).

Comparing the two study groups regarding PIV, NLR, PLR, and MLR, all variables showed significant difference as shown in **Table 4**. As presented in **Table 5**, PIV showed significant positive correlation with BDCAF and E.S.R (p= 0.025 and 0.039, respectively). None of the investigated haematological markers showed significant correlation with BODI.

**Table 4.** Blood cells derived ratios among the study groups.

**Variable**	**BD patients (n=40)**	**Controls (n=40)**	**P value**
**PIV**	230.01 (45.03–1383.2)	158.63 (55.6–425.53)	0.04
**NLR**	1.58 (0.64–5.07)	1.36 (0.75–1.93)	0.04
**PLR**	153.4 (78.57–337.69)	107.1 (72.11–200)	< 0.001
**MLR**	0.23 (0.08–0.62)	0.19 (0.07–0.4)	0.007

Data expressed as mean± SD/ median (range). The P-value was significant if < 0.05. BD: Behcet disease; PIV: pan immune inflammation value; NLR: neutrophils to lymphocytes ratio; PLR: platelets to lymphocytes ratio; MLR: monocyte to lymphocyte ratio.

**Table 5.** Correlation of clinical and laboratory characteristics with studied variables.

**Variable**	**PIV**		**NLR**		**PLR**		**MLR**	
	**R**	**P**	**R**	**P**	**R**	**P**	**R**	**P**
**Age**	0.16	0.34	−0.12	0.48	−0.09	0.6	0.05	0.7
**Disease duration**	−0.02	0.89	0.01	0.95	−0.27	0.09	−0.005	0.98
**BDCAF**	0.354	0.025	0.2	0.22	0.3	0.046	0.2	0.19
**BODI**	−0.25	0.13	0.085	0.6	−0.06	0.7	−0.2	0.3
**ESR**	0.33	0.039	0.14	0.39	0.28	0.08	0.1	0.26

The P-value was significant if < 0.05. R: correlation coefficient; PIV: pan immune inflammation value; NLR: neutrophils to lymphocytes ratio; PLR: platelets to lymphocytes ratio; MLR: monocyte to lymphocyte ratio; BODI: Bechet’s syndrome overall damage index; BDCAF: Behcet’s Disease Current Activity Form.

**Figure 1.**
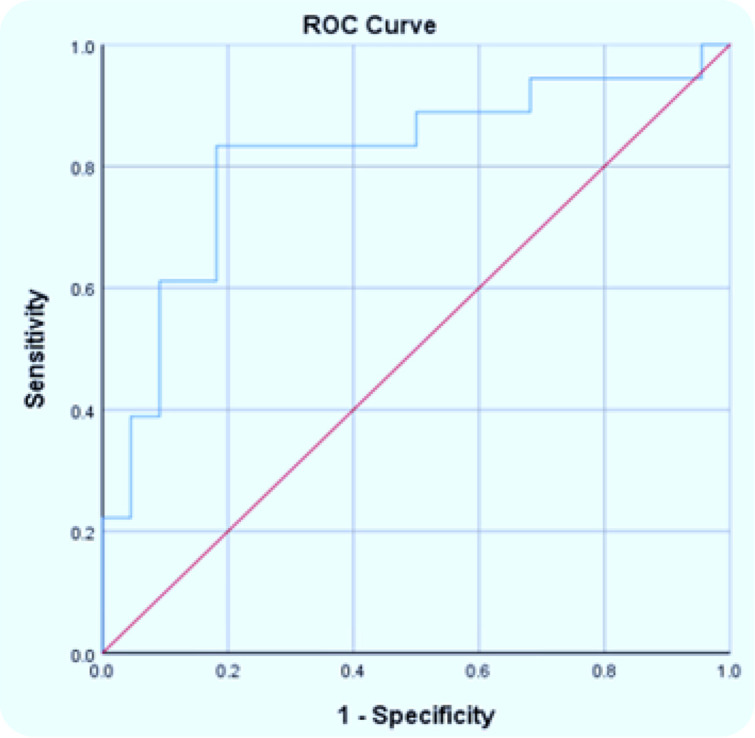
The receiver operating characteristic (ROC) curve analysis revealed that, at a cutoff value of 238.26 for detecting active ocular involvement, the area under the curve was 0.81 (95% CI, p=0.001). PIV exhibited a sensitivity of 83%, specificity of 82%, and an accuracy of 82.5%.

We analysed the differences in the studied blood derived ratios between patients with active and inactive systemic involvement, and we detected significant association of ocular and musculoskeletal involvement with high level of PIV (p= 0.001 and 0.037, respectively) as observed in **Table 6**. The use of biological therapy, specifically anti-TNF α agents, did not have a statistically significant impact on PIV, NLR, PLR, and MLR (p= 0.78, 0.16, 0.73 and 0.32, respectively). The ROC curve analysis revealed that, at a cutoff value of 238.26 for detecting active ocular involvement, the area under the curve was 0.81 (95% CI, p=0.001). PIV exhibited a sensitivity of 83%, specificity of 82%, and an accuracy of 82.5% as shown in **Figure 1**.

**Table 6.** Comparison of studied variables according to the presence of active system involvement in BD patients.

**Active system involvement**	**N**	**PIV**		**NLR**		**PLR**		**MLR**	
		**Median (range)**	**P**	**Median (range)**	**P**	**Median (range)**	**P**	**Median (range)**	**P**
**Ocular**									
**Yes**	18	336.12 (46.95–1383.2)	**0.001**	1.69 (0.75–5.07)	**0.3**	148.8 (78.6–337.69)	**0.7**	0.27 (0.1–0.62)	**0.03**
**No**	22	152.9 (45.03–589.6)		1.69 (0.75–5.07)		154.3 (92.9–311.8)		0.2 (0.08–0.49)	
**Musculoskeletal**									
**Yes**	7	602.95 (108.1–1383.2)	**0.037**	1.66 (0.99–5.07)	**0.7**	150.9 (78.6–291.2)	**0.7**	0.35 (0.16–0.62)	**0.016**
**No**	33	197.3 (45–589.6)		1.56 (0.64–4.69)		155 (80–337.7)		0.21 (0.08–0.44)	
**Neurological**									
**Yes**	7	154.7(45.03–1383.2)	**0.2**	1.25 (0.64–5.07)	**0.24**	151 (115–311.8)	**0.5**	0.23 (0.08–0.47)	**0.8**
**No**	33	242.2 (54.6–790)		1.63 (0.79–4.69)		153.4 (78.57–337.7)		0.23 (0.1–0.62)	

The P-value was significant if < 0.05. NLR: neutrophils to lymphocytes ratio; PLR: platelets to lymphocytes ratio; MLR: monocyte to lymphocyte ratio; PIV: pan immune inflammation value.

## DISCUSSION

BD is a persistent autoinflammatory multisystemic vasculitis. An aberrant immune response is accused of initiating BD in a genetically predisposed person. Major pathological features include neutrophil hyperfunction and vasculitis. Systemic inflammation is linked to changes in circulating blood cell composition.^19,20^ Though numerous markers were linked to disease activity in BD, no definitive laboratory test has been identified. However, recognition of new laboratory markers is mandatory to assist anticipating the disease course and avoid complications.

While previous studies, including those published in the Mediterranean Journal of Rheumatology (MJR), have focused on novel haematological markers and ratios in rheumatological disorders, our study is among the first to evaluate the possible clinical value of the PIV in BD. Furthermore, our study provides new insights relevant to BD patients in the Middle East. A previous publication in MJR reported that NLR and PLR might act as good predictors of paediatric systemic lupus erythematosus (SLE) disease activity.^21^ Another study highlighted haematological abnormalities in Sjogren syndrome.^22^

The current study detected significantly higher PIV, NLR, PLR and MLR in BD patients than in healthy participants. PIV showed significant positive correlation with BDCAF and ESR. We detected a significantly greater PIV in patients with active ocular and musculoskeletal disease. At a cutoff of 238.26, PIV yielded an AUC of 0.81 (95% CI, p=0.001), with 83% sensitivity and 82% specificity for detecting active ocular involvement.

PIV is a composite haematological marker based on the neutrophil, platelet, monocyte and lymphocyte counts. ^8^ Our study showed that both disease activity and ESR were positively correlated with PIV in BD patients. Lee and colleagues concluded that a higher PIV level at diagnosis was linked to a reduced survival rate in patients with antineutrophil cytoplasmic antibody associated vasculitis.^10^ Furthermore, Başaran et al. observed a significantly higher PIV in RA patients with high disease activity.^23^

Regarding specific system involvement in BD patients, we detected a significantly higher PIV and MLR in patients with active ocular and musculoskeletal involvement than those without active ocular and musculoskeletal involvement, respectively. Ocak et al., reported that PIV is a positive predictor for vascular BD.^24^

PIV demonstrated a positive correlation with disease activity in addition to its good diagnostic performance in distinguishing patients with active ocular involvement, as shown by the ROC curve analysis with (83% and 82%) sensitivity, and specificity, respectively. These findings may be attributed to its inclusion of neutrophils, platelets, monocytes and lymphocytes. These cells collectively mediate vascular inflammation. Neutrophils contribute through the release of neutrophil extracellular traps, which are prothrombotic, while platelet-derived microparticles aggravate endothelial injury in vasculitis.^25,26^ Concurrently, monocytes promote sustained inflammatory responses by producing cytokines.^27^ These cytokines significantly enhance adhesion between neutrophils and endothelial cell.^28^ Angiogenic T lymphocytes play a role in maintaining vascular integrity so, their deficiency in active BD patients, as observed by Kul et al., has a pivotal rule in the pathogenesis of BD.^29^ By encompassing this cellular combination, PIV may offer a more comprehensive reflection of the complex immune mechanisms underlying Behçet’s-related disease activity and ocular involvement than isolated haematological indices.

Mentesoglu and colleagues investigated the systemic immune-inflammation index (SII) in BD, that is calculated from platelet, neutrophil and lymphocyte counts. They observed a significantly higher SII in patients with active BD than those with inactive disease (p<0.001),^30^ which agrees with our observation that PIV is positively correlated with disease activity.

Neutrophil hyperactivity-induced innate immune system dysfunction is proposed to be a core attribute of the pathophysiology of BD.^31^ We observed a significantly greater NLR in BD patients compared to the controls however, we could not find an association between NLR and BDCAF score. Our results agree with Alan et al.,^32^ who found a significantly high NLR in BD patients compared to healthy controls without correlation with BD severity.

The lack of correlation between NLR and BDCAF in our study contrasts findings by Changfen et al.,^33^ potentially due to differences in disease duration or treatment regimens. For instance, 80% of our patients received corticosteroids, which may affect neutrophil and lymphocyte counts.^34^

Platelets are activated in systemic vasculitis, producing microparticles that change vascular function and accelerate the progression of vasculitis by inducing a procoagulant and pro-inflammatory state.^26^ Our study demonstrated an elevated PLR in the BD group compared to controls, which agrees with the previous reports.^32,35^ Additionally, our study detected a positive correlation between PLR and BDCAF that aligns with a meta-analysis which concluded that patients with active BD had a considerably higher PLR than those with inactive disease (p = 0.005).^35^A previous study found that high PLR was linked to disease activity in polymyositis patients,^36^ which aligns with our results.

Monocytes are one of the main producers of oxidative and proinflammatory cytokines.^37^ Current results revealed a higher MLR in BD patients than in controls, which aligns with Huang and colleagues.^13^ In addition, they observed a positive correlation between MLR and ESR (r=0.363, p<0.05) which disagrees with our observation. Tezcan et al.^11^ found that patients with BD had considerably lower lymphocyte to monocyte ratio than the control group (p= 0.021). An elevated MLR in patients presenting with active musculoskeletal and ocular manifestations may indicate sustained monocyte-mediated inflammation.

The limitations of our study include the small sample size, which reduces generalisability and the limited number of patients not receiving corticosteroid therapy, which restricts our ability to explore the effect of corticosteroids on the studied haematological ratios.

## CONCLUSION AND RECOMMENDATIONS

Both PIV and PLR show significant positive correlation with disease activity. In addition, PIV has good diagnostic performance for active ocular involvement in BD in comparison to other studied blood cells derived ratios. We recommend incorporating PIV into the routine clinical evaluation of patients with BD, especially in cases where ocular involvement is suspected. Prospective longitudinal studies on a larger scale are warranted to determine whether repeated PIV assessments can help predict treatment outcomes or anticipate disease flares in BD, in addition to assessing the impact of medications on haematological ratios and indices.

## Data Availability

The datasets generated and/or analysed during the study are available from the corresponding author upon reasonable request.
